# In Situ Holographic Monitoring of Stress Corrosion Dynamics of Alloy 625 in Cl^−^ + S_2_O_3_^2−^ Solution

**DOI:** 10.3390/molecules31101716

**Published:** 2026-05-18

**Authors:** Pengyu Yang, Yunzhou Gu, Fuli Wu, Boyu Yuan, Liang Li, Chao Wang

**Affiliations:** 1 School of Chemistry & Materials Science, Jiangsu Normal University, Xuzhou 221116, China; 2020251678@jsnu.edu.cn (P.Y.); wangc@jsnu.edu.cn (C.W.); 2 School of Physics and Electronic Engineering, Jiangsu Normal University, Xuzhou 221116, Chinayuanby@jsnu.edu.cn (B.Y.)

**Keywords:** stress corrosion, digital holography, intergranular corrosion, alloy 625

## Abstract

This study examined the stress corrosion of Alloy 625 in Cl^−^ + S_2_O_3_^2−^ solutions using digital holography in combination with electrochemical methods. Without elastic tensile stress, intergranular corrosion (IGC) occurred, due to the higher activity of grain boundaries compared to the grain interior and to preferential adsorption of sulfur (produced by S_2_O_3_^2−^ decomposition) at these boundaries. Digital holography observations showed that IGC initiated at certain grain boundaries and propagated to adjacent boundaries, even in the absence of elastic tensile deformation. Applying elastic tensile stress (260 MPa, ~46% σy) increased the defect density within the oxide film, thereby enhancing corrosion and anodic currents, and inducing river-like cracks. Although elastic tensile stress suppressed IGC, it simultaneously promoted stress corrosion cracking (SCC), as the stress exerted a stronger accelerating effect on corrosion than the grain-boundary did. Digital holography allowed in situ monitoring of the stress corrosion process in Alloy 625, demonstrating that cracks initiated via localized corrosion/IGC and subsequently propagated along the direction of the applied stress.

## 1. Introduction

Nickel-based Alloy 625 is a high-performance alloy primarily composed of nickel and strengthened mainly through the addition of chromium, molybdenum, and niobium. A chromium content of 20.0–23.0% provides excellent oxidation and corrosion resistance, while a molybdenum content of 8.0–10.0% enhances corrosion resistance in reducing environments and improves strength. It exhibits superior corrosion resistance, high strength, resistance to high-temperature corrosion, excellent weldability, outstanding oxidation resistance, and good fatigue performance and workability. It is widely utilized in petrochemical, marine, nuclear power, and aerospace applications. However, under combined elastic tensile stress and aggressive ions (e.g., Cl^−^), Alloy 625 is susceptible to intergranular corrosion (IGC) [1], uniform corrosion, pitting corrosion [2], and stress corrosion cracking (SCC). Corrosion of metallic materials poses a critical industrial problem [3,4]. SCC is a primary failure mode for nickel-based alloys, necessitating detailed investigation of Alloy 625 under these conditions.

Zhou et al. [5] investigated sulfide stress cracking (SSCC) of X60 pipeline steel/Alloy 625 welded joints in H_2_S environments using constant load testing, weight loss measurement, and finite element analysis. Their results revealed high SSCC susceptibility and hydrogen-embrittlement-accelerated crack propagation along the fusion boundaries. Tsujikawa et al. [6] employed slow strain rate testing (SSRT) to demonstrate that thiosulfate ions (S_2_O_3_^2−^) can effectively simulate H_2_S-induced stress corrosion cracking in corrosion-resistant alloys (CRAs) and SSCC in high-strength low-alloy steels, supporting the use of S_2_O_3_^2−^ as a surrogate for H_2_S. Xia et al. [7] emphasized the critical role of sulfur in the corrosion of nickel-based alloys by analyzing E-pH diagrams, potential-pH diagrams, and species distribution curves. They pointed out that S_2_O_3_^2−^, which predominates in neutral/alkaline environments, decomposes into elemental sulfur (S) and sulfate (SO_4_^2−^) (Equation (1)). The resulting sulfur adsorbs onto the alloy surface, weakening metallic bonds and hindering oxide formation by blocking water adsorption [8], thereby accelerating anodic dissolution:

2H^+^ + 3S_2_O_3_^2−^ → 4S + 2SO_4_^2−^ + H_2_O  ΔG = −169.764 kJ (20 °C) (1)

Extensive research exists on SCC of nickel-based alloys in Cl^−^ + S_2_O_3_^2−^ solutions. Gorman [9] identified SSCC as an SCC submode in pressurized water reactors, attributing it primarily to low-valence sulfur (e.g., S_2_O_3_^2−^), while high-valence species (e.g., SO_4_^2−^) did not accelerate SCC in high-pH environments. Divi et al. [10] found that annealed Alloy 625 resisted SCC in high-chloride environments but became susceptible after heavy cold forming. Was et al. [11] used constant-extension-rate tensile experiments to report severe intergranular SCC (IGSCC) in Alloy 625 in supercritical water, attributed to its thin, Cr-rich inner/Fe-rich outer oxide layer. Liu et al. [12] observed a linear increase in SSCC sensitivity of X80/Inconel 625 welds with Na_2_S_2_O_3_ concentration (10^−4^–10^−2^ M), which diminished at 10^−1^ M. Rodriguez et al. [13,14] identified SCC susceptibility in sensitized Alloy 601 (0.1 M Na_2_S_2_O_3_) and Alloy 690 (0.01 M Na_2_S_2_O_3_, 90 °C), linking the latter to chromium depletion from carbide precipitation by using SSRT and the electrochemical method. Luo et al. [15] showed that both tensile and compressive stresses increased localized surface reactivity of Alloy 800 in thiosulfate solutions, as measured by scanning electrochemical microscopy (SECM). Tsai and Chou [16] used loading frequency to find transgranular fatigue crack growth in Alloy 690 but intergranular cracking in sensitized Alloy 600 in thiosulfate solutions. Yang et al. [17] attributed the lower caustic SCC susceptibility of Alloy 690 compared with Alloy 800 to differences in passive film stability, with S_2_O_3_^2−^ destabilizing films and enhancing anodic dissolution of Ni, as evidenced by electrochemical method measurements and Auger electron spectroscopy (AES). Tian et al. [18] reported hydrogen embrittlement-dominated SCC at low S_2_O_3_^2−^ (10^−4^ M) and pitting-dominated SCC at high S_2_O_3_^2−^ (10^−2^ M) in E690 steel by using the electrochemical method and electron back-scattered diffraction (EBSD).

Despite these studies, the complexity of stress corrosion dynamics and variable sulfur valence states necessitates further investigation of Alloy 625 in S_2_O_3_^2−^-containing environments. Traditional electrochemical techniques, such as voltammetry, electrochemical impedance spectroscopy (EIS), and electrochemical noise (EN), can only provide macroscopic corrosion information [19] and are unsuitable for capturing details of localized corrosion. Surface physical analysis methods, such as scanning electron microscopy (SEM) and X-ray photoelectron spectroscopy (XPS), can characterize the morphology and composition of electrodes before and after corrosion, but they cannot track dynamic changes during the corrosion process and thus fail to capture the evolving nature of stress corrosion cracking (SCC).

Various in situ techniques are available for studying corrosion processes in metallic materials. For example, real-time optical microscopy is non-destructive, provides visual information, and allows dynamic monitoring; however, it is constrained by the diffraction limit, and corrosion products on the electrode surface can interfere with light reflection, thereby reducing resolution. Acoustic emission technology excels at detecting internal “micro-earthquakes” (i.e., damage events) in operating structures, recording their occurrence, location, and activity level. Nevertheless, its signals are susceptible to interference and are sensitive to material properties and wave propagation paths. Digital image correlation (DIC) enables full-field, non-contact deformation measurement, but its accuracy heavily depends on speckle pattern quality, illumination conditions, and computational stability. Digital holography offers key advantages, including a non-destructive working principle, rapid response, and high system performance. Phase shifts in the object beam can reveal subtle specimen features for quantitative analysis. Although its maximum lateral resolution remains diffraction-limited, the technique enables in situ observation of dynamic processes and localized corrosion in microscale systems, and can even extract diffusion-layer thickness from phase-distribution data.

In summary, each technique has its distinct strengths and limitations and is best applied within an appropriate scope. In situ methods such as digital holography [20,21,22,23], offering high spatiotemporal resolution, are particularly suitable for stress corrosion studies. For example, Wu et al. [23] used digital holography to observe stress-induced crack initiation and propagation in Alloy 625 in a NaCl environment. In the present work, a significantly lower tensile stress (260 MPa) is applied. The stress corrosion behavior of Alloy 625 in thiosulfate-containing environments remains underexplored with in situ techniques. Therefore, this study aims to fill that gap by systematically examining stress-assisted corrosion processes using coupled electrochemical and holographic monitoring.

Due to the inherent complexity of stress corrosion, in situ investigation is essential. To the best of our knowledge, however, few studies have examined the stress corrosion behavior of Alloy 625 with in situ approaches such as digital holography. This work combines electrochemical methods with in situ digital holography to study the stress corrosion processes of Alloy 625 in Cl^−^ + S_2_O_3_^2−^ solutions under elastic tensile stress. The obtained phase maps (from digital holography), surface analyses (SEM, EDS), and electrochemical data are used to elucidate the underlying corrosion mechanism under stress.

## 2. Results

### 2.1. Tafel Curves

**Figure 1:**
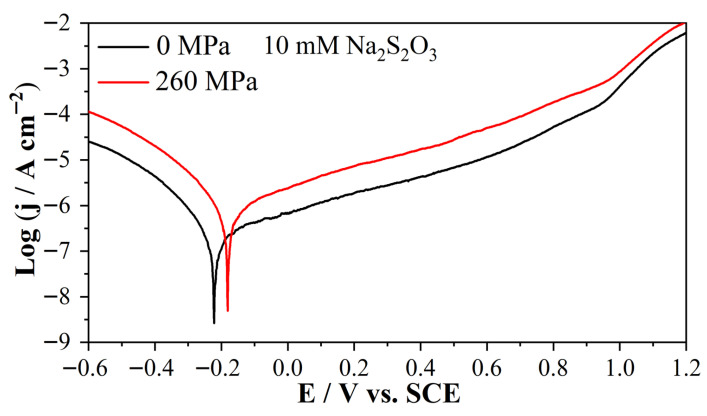
Tafel plots of 625 alloy in 0.5 M NaCl + 10 mM Na_2_S_2_O_3_ solution, without and with an elastic tensile stress (260 MPa, ~46% σy), scan rate 1 mV/s.

**Figure 2:**
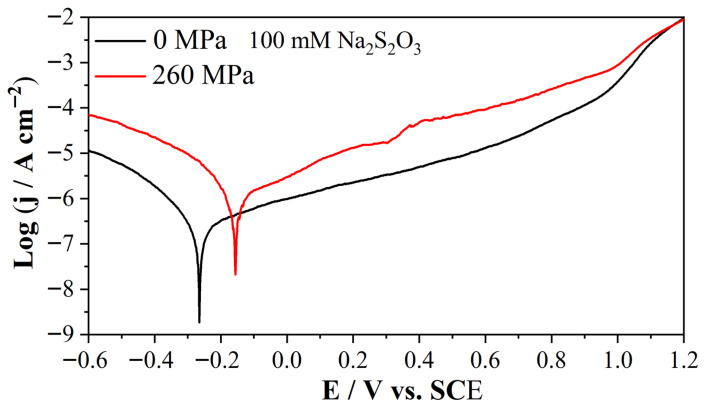
Tafel plots of 625 alloy in 0.5 M NaCl + 100 mM Na_2_S_2_O_3_ solution, without and with an elastic tensile stress (260 MPa, ~46% σy), scan rate 1 mV/s.

Figure 1 shows the Tafel curves of Alloy 625 in 0.5 M NaCl + 10 mM Na_2_S_2_O_3_ solution (pH = 5.8) with and without the elastic tensile stress (260 MPa, ~ 46% σy). Figure 2 shows the Tafel curve of Alloy 625 in 0.5 M NaCl + 100 mM Na_2_S_2_O_3_ solution (pH = 6.09) with and without the elastic tensile stress (260 MPa, ~ 46% σy). As shown in Figure 1 and Figure 2, the elastic tensile stress increases both cathodic and anodic currents. The corresponding corrosion parameters of Figure 1 and Figure 2 are shown in Table 1. As shown in Table 1, the elastic tensile stress consistently increases corrosion current (j_corr_) and shifts corrosion potential (E_corr_) positively. Under stress, j_corr_ increases with S_2_O_3_^2−^ concentration, whereas the opposite occurs without stress due to the higher cathodic current in solution with higher S_2_O_3_^2−^ concentration.

### 2.2. Potentiodynamic Polarization Curves and the Corresponding Phase Maps

**Figure 3:**
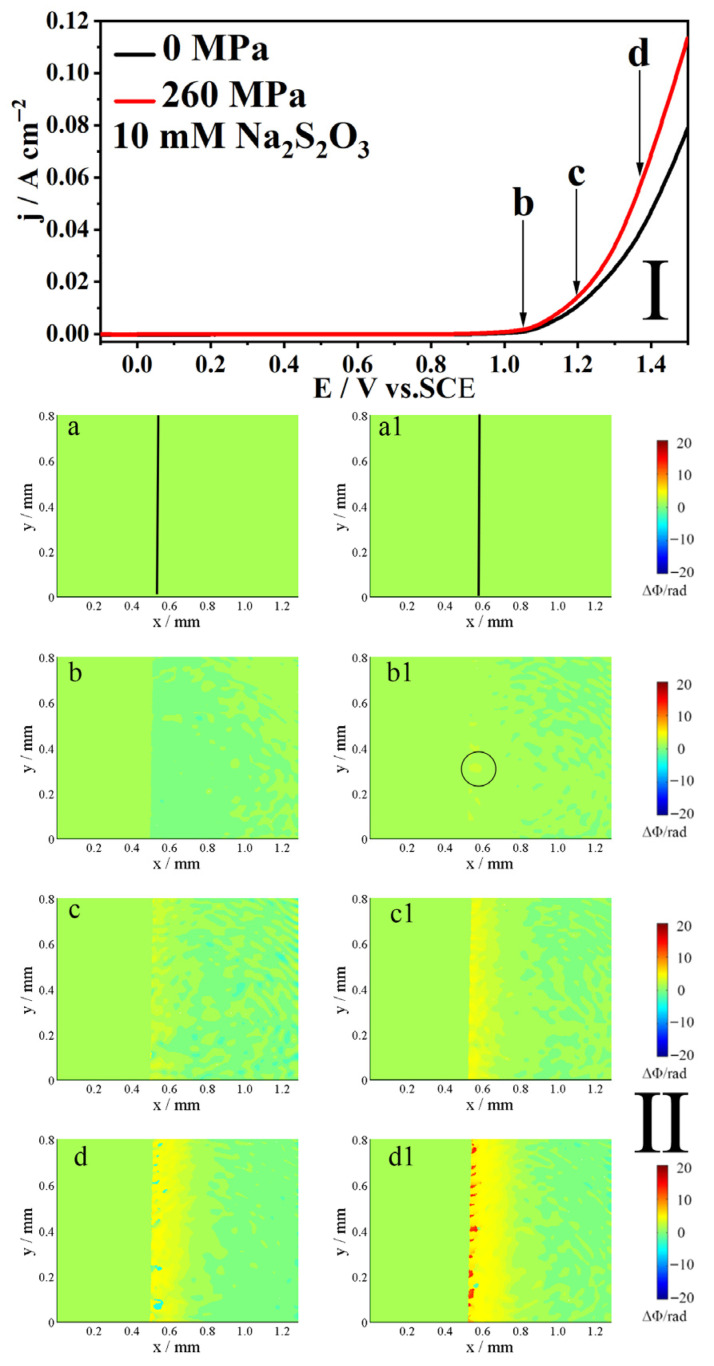
The polarization curves (**I**) of 625 alloy in 0.5 M NaCl + 10 mM Na_2_S_2_O_3_ solution and the corresponding phase maps (**II**) at different potentials to points b–d in the polarization curves, (**a**,**a1**) obtained at the open circuit potential (the same below), without and with an elastic tensile stress, scan rate 10 mV/s.The line in (**a**,**a1**) indicates the interface. The circled area in (**b1**) marks the region of localized dissolution. The (**b**–**d**,**b1**–**d1**) in Part **II** correspond to points b–d in Part **I**.

Figure 3 shows the potentiodynamic polarization curve (I) and the corresponding phase maps (II) of Alloy 625 in 0.5 M NaCl + 10 mM S_2_O_3_^2−^ solution with and without the elastic tensile stress. Since the applied potential is much higher than the corrosion potential, the corrosion process is predominantly anodic. In this work, the anodic process involves the dissolution of the electrode; hence, it is referred to as anodic dissolution. As shown in Part I of Figure 3, the stress has a minimal effect on the anodic current below 0.9 V vs. SCE. Above 0.9 V, the stress significantly increases the current, enhancing anodic dissolution.

**Figure 4:**
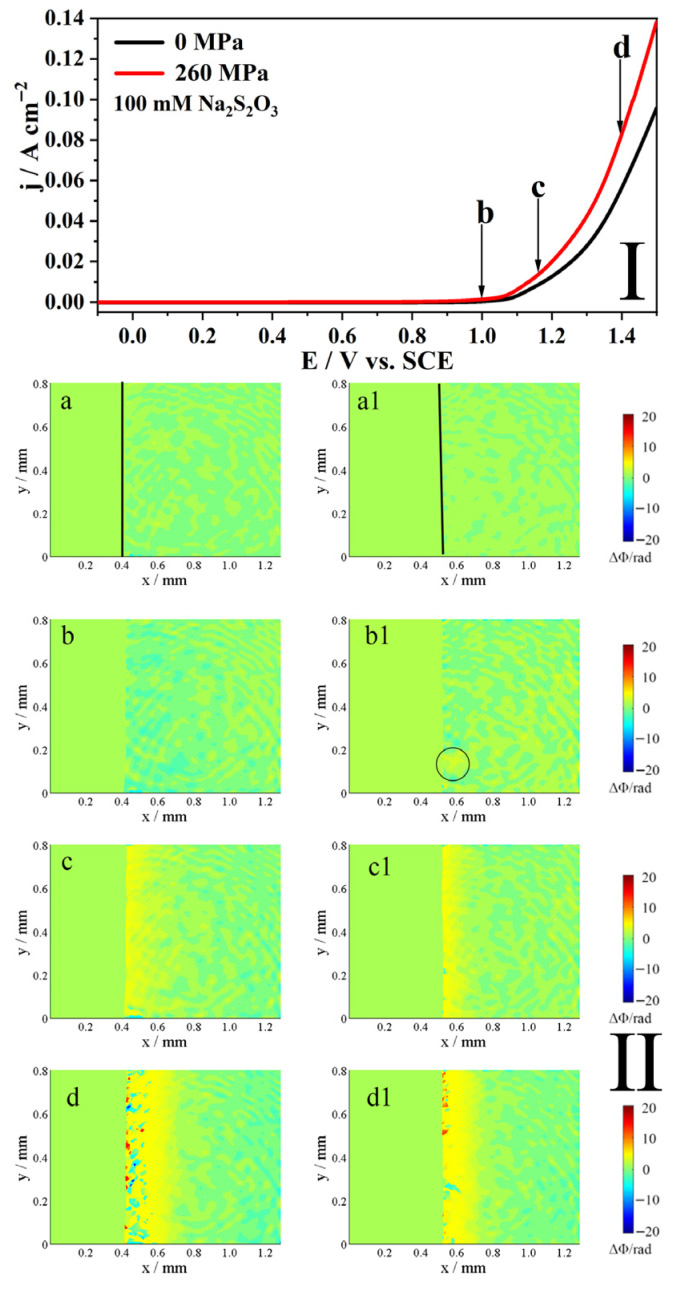
The polarization curves (**I**) of 625 alloy in 0.5 M NaCl + 100 mM Na_2_S_2_O_3_ solution and the corresponding phase maps (**II**) at different potentials to points b–d in the polarization curves, without and with an elastic tensile stress, scan rate 10 mV/s. The line in (**a**,**a1**) indicates the interface. The circled area in (**b1**) marks the region of localized dissolution. The (**b**–**d**,**b1**–**d1**) in Part **II** correspond to points b–d in Part **I**.

**Figure 5:**
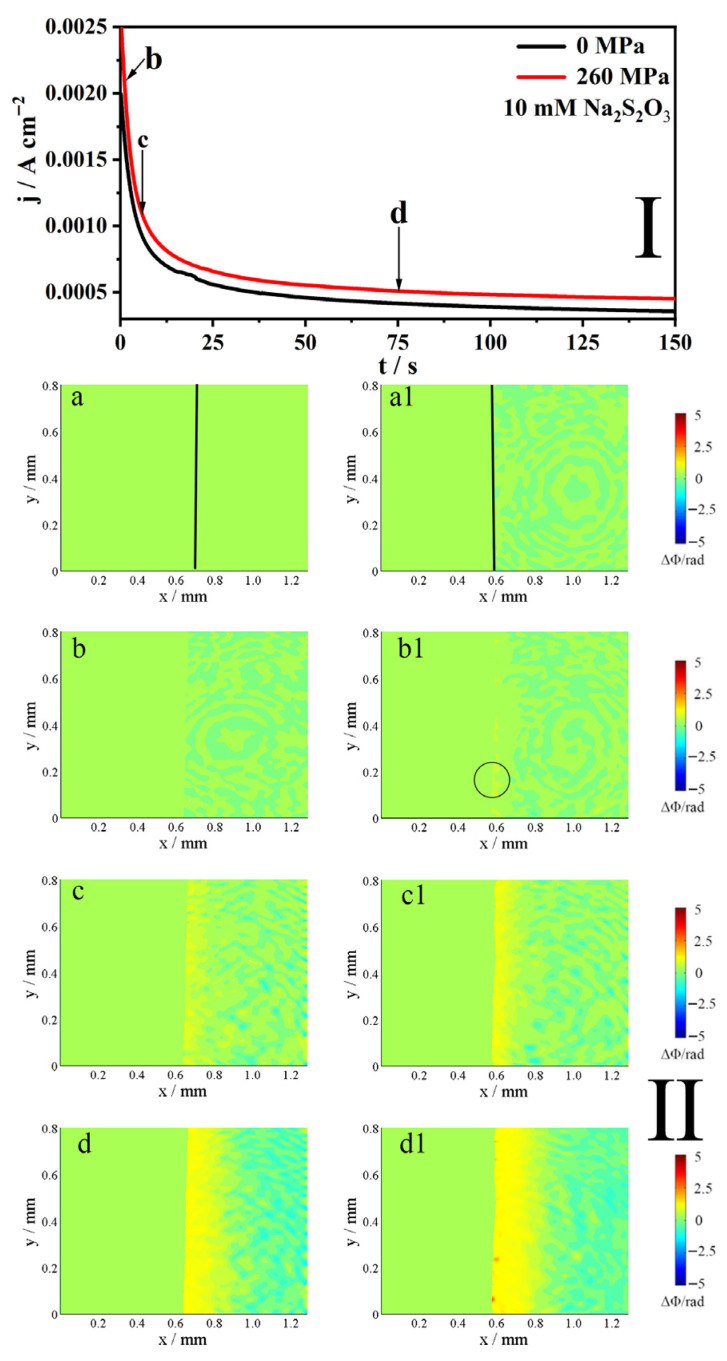
The j-t curves (**I**) of 625 alloy in 0.5 M NaCl + 10 mM Na_2_S_2_O_3_ solution at 1.0 V, with and without the elastic tensile stress, and the phase maps (**II**) at different times corresponding to points b–d in the j-t curves, respectively. The line in (**a**,**a1**) indicates the interface. The circled area in (**b1**) marks the region of localized dissolution. The (**b**–**d**,**b1**–**d1**) in Part **II** correspond to points b–d in Part **I**.

**Figure 6:**
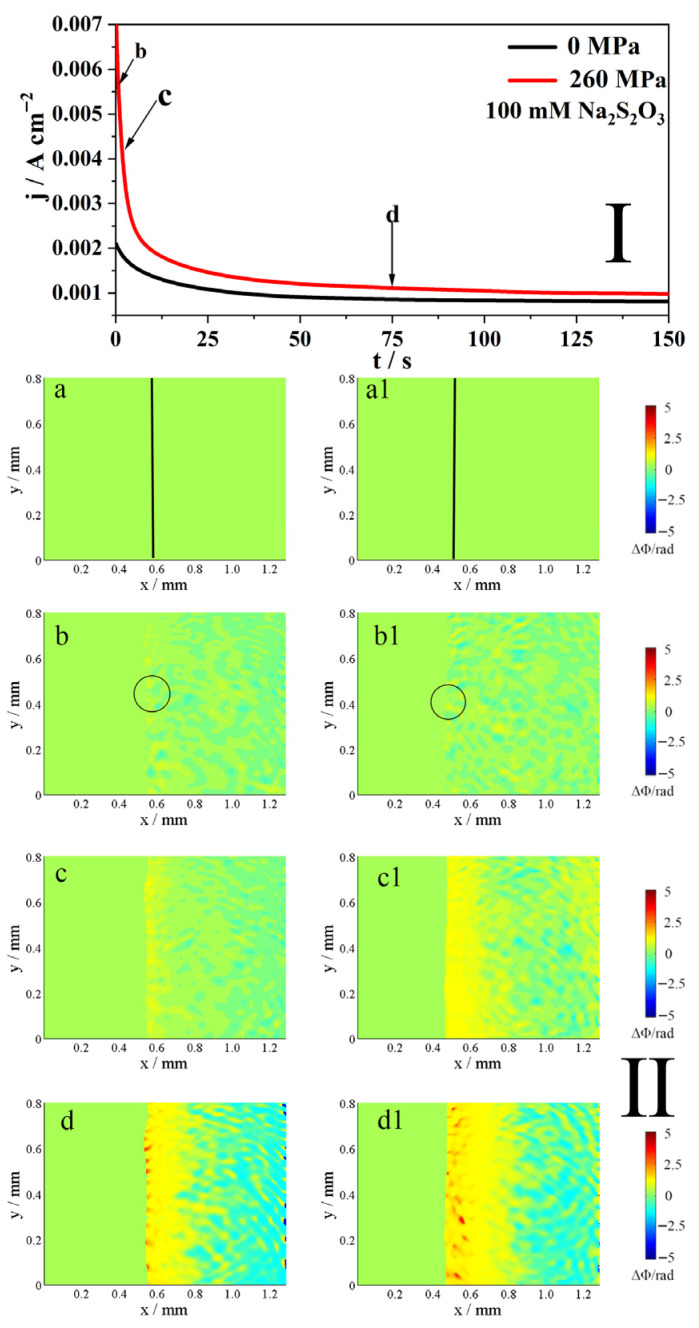
The j-t curves (**I**) of 625 alloy in 0.5 M NaCl + 100 mM Na_2_S_2_O_3_ solution at 1.0 V, with and without the elastic tensile stress, and the phase maps (**II**) at different times corresponding to points b–d in the j-t curves, respectively. The line in (**a**,**a1**) indicates the interface. The circled areas in (**b**,**b1**) mark the region of localized dissolution. The (**b**–**d,b1**–**d1**) in Part **II** correspond to points b–d in Part **I**.

The phase maps in Figure 3(a,a1) were obtained at the open circuit potential (similarly, hereinafter, Figure 4(a,a1), Figure 5(a,a1), and Figure 6(a,a1)). The interface between the electrode (left) and the electrolyte (right) is marked by a black line. The variation in concentration (Δc) primarily refers to the change in the concentration of the soluble species at the electrode|electrolyte interface during anodic dissolution processes. The color of the map corresponds to the local concentration change (Δc) as follows:

Green: No change in concentration (Δc = 0, ΔΦ = 0).

Blue: Decrease in concentration (Δc < 0, ΔΦ < 0).

Red/Yellow: Increase in concentration (Δc > 0, ΔΦ > 0).

As depicted in the phase maps of Figure 3(II), the potential increase to point b reveals the emergence of local yellow areas at the electrode|electrolyte interface under the elastic tensile stress (Figure 3(b1)). This indicates a rise in local concentration and the occurrence of localized corrosion. Conversely, the interface without the elastic tensile stress remained unchanged except for noise (Figure 3b), suggesting that anodic dissolution had not commenced. When the potential was shifted positively to point c, a shallow yellow area appeared on the unstressed interface (Figure 3c), indicating the onset of anodic dissolution. Under the influence of the elastic tensile stress, the interfacial concentration is higher than that in the unstressed condition (Figure 3(c1)). At point d, red areas appear within the yellow regions of the stressed interface (Figure 3(d1)), signifying that the elastic tensile stress further exacerbates anodic dissolution. The unstressed interface also shows an expanded yellow area (Figure 3d), indicating that anodic dissolution is further strengthened as the potential increases. In summary, elastic tensile stress increases Alloy 625′ susceptibility to localized corrosion at lower potentials, raises the interfacial ion concentration, and thereby accelerates its anodic dissolution.

Figure 4 shows the potentiodynamic polarization curve (I) and the corresponding phase maps (II) of Alloy 625 in 0.5 M NaCl + 100 mM S_2_O_3_^2−^ solution with and without the elastic tensile stress. As depicted in Part I, the stress minimally affects the current at potentials below 0.9 V. However, at higher potentials, it significantly increases the anodic current, enhancing the anodic dissolution of Alloy 625.

The phase maps in Part II of Figure 4 provide a visual representation of this process. When the potential is increased to point b, the interface without the elastic tensile stress exhibits only noise and no anodic dissolution (Figure 4b). In contrast, under the elastic tensile stress, a local yellow area appears on the interface (Figure 4(b1)), indicating that the local corrosion has begun. At point c, a continuous yellow area signifying the commencement of active dissolution is observed on both interfaces, with and without stress (Figure 4(c,c1)). As the potential reaches point d, active dissolution occurs on both electrodes, regardless of the applied stress (Figure 4(d,d1)).

### 2.3. The j-t Curves and the Corresponding Phase Maps

Figure 5 shows the j-t curve (I) and the corresponding phase maps (II) of Alloy 625 at E = 1.0 V in 0.5 M NaCl + 10 mM S_2_O_3_^2−^ solution, with and without an elastic tensile stress. As shown in Part I of Figure 5, the current initially decreases before gradually stabilizing over time, regardless of the applied stress. Elastic tensile stress elevated the current (t = 130 s) by 25% in the 10 mM S_2_O_3_^2−^ solution.

As depicted in the phase maps of Figure 5(II), the interface evolves dynamically over time. At the initial stage (point b), the application of the elastic tensile stress induces local corrosion, as highlighted in Figure 5(b1); in contrast, the interface without deformation remains unchanged, exhibiting only background noise (Figure 5b). Upon reaching point c, a diffusion layer becomes evident at the interface under both stressed and unstressed conditions; however, this layer is substantially thicker when the elastic tensile stress is applied (Figure 5(c,c1)). At the final stage (point d), the interface under both conditions develops a distinct two-layer structure, defined by an increase in concentration within the inner layer and a corresponding decrease in the outer layer (Figure 5(d,d1)).

Figure 6 shows the j-t curve (I) and the corresponding phase maps (II) of Alloy 625 at E = 1.0 V in a 0.5 M NaCl + 100 mM S_2_O_3_^2−^ solution, with and without the elastic tensile stress. As depicted in Part I of Figure 6, the current initially decreases and then gradually reaches a steady state, regardless of the applied stress. The application of the elastic tensile stress increases this steady-state current (t = 130 s) by 21.6% according to the j-t curves in Figure 6. In comparison to the results in the 10 mM S_2_O_3_^2−^ solution (Part I in Figure 5), the higher S_2_O_3_^2−^ concentration leads to an increased current under both stressed and unstressed conditions.

According to the phase maps in Part II of Figure 6, the interface evolves differently depending on the presence of elastic tensile stress. Initially, at point b, stress-induced local corrosion (indicated by black circles) is observed in both cases, with and without elastic tensile stress (Figure 6(b,b1)). As polarization continues to point c, a diffusion layer develops at the interface. The application of elastic tensile stress significantly accelerates the thickening of this layer (Figure 6(c,c1)). Finally, upon reaching point d, the interface in both scenarios exhibits a distinct two-layer structure: an inner layer where concentration increases and an outer layer where it decreases (Figure 6(d,d1)). Furthermore, the elastic tensile stress causes substantial increases in the thickness of the inner layer, as indicated by the phase maps in Figure 6.

### 2.4. Surface Analysis

#### 2.4.1. Morphology

**Figure 7:**
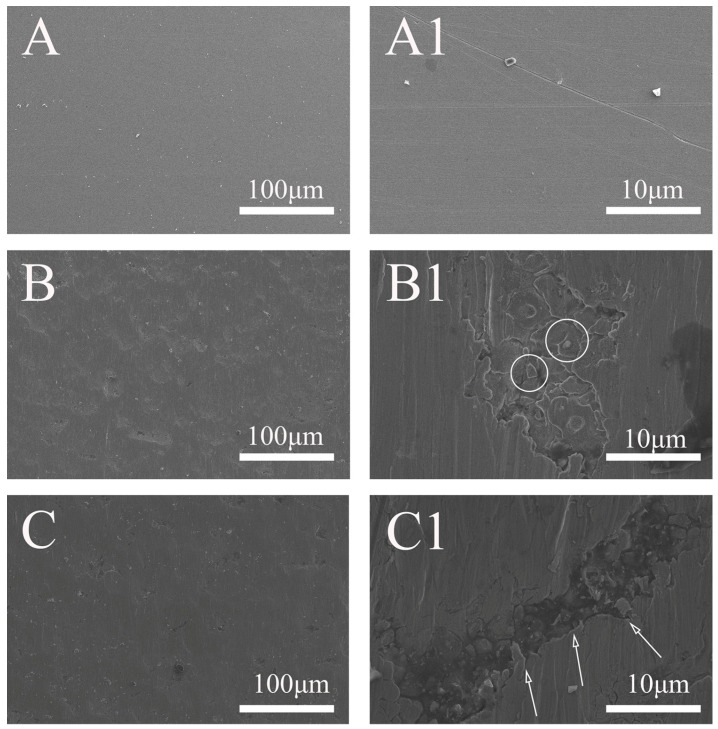
Surface morphologies of 625 alloy recorded before (**A**,**A1**) and after potentiostatic polarization at 1.0 V for 150 s in 0.5 M NaCl + 10 mM Na_2_S_2_O_3_ solutions without (**B**,**B1**) and with the elastic tensile stress (**C**,**C1**).

Figure 7 presents the surface morphologies of Alloy 625 before and after anodic dissolution at 1.0 V for 150 s in a 0.5 M NaCl + 10 mM S_2_O_3_^2−^ solution, comparing the effects with and without the elastic tensile stress (corresponding to Figure 5). Initially, the surface was smooth (Figure 7A), with only polishing scratches visible upon magnification (Figure 7(A1)). After anodic dissolution, without the elastic tensile stress, corrosion products formed on the surface (Figure 7B), and magnification revealed the underlying IGC (Figure 7(B1)). In contrast, when the elastic tensile stress was applied, the surface also exhibited corrosion products (Figure 7C), but magnification uncovered a distinct river-like crack (Figure 7(C1)) with severe corrosion within it. Notably, the crack orientation was consistent with the direction of the applied stress.

**Figure 8:**
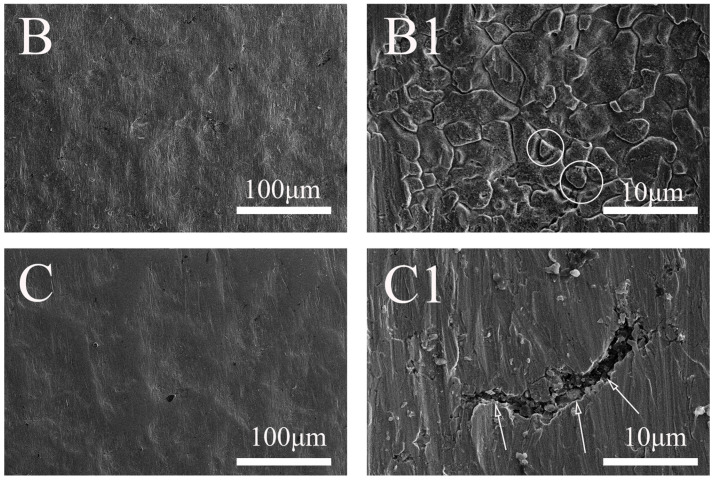
Surface morphologies of 625 alloy recorded after potentiostatic polarization at 1.0 V for 150 s in 0.5 M NaCl + 100 mM Na_2_S_2_O_3_ solutions, without (**B**,**B1**) and with the elastic tensile stress (**C**,**C1**).

Figure 8 shows the surface morphologies of Alloy 625 after anodic dissolution in a 0.5 M NaCl + 100 mM S_2_O_3_^2−^ solution, comparing conditions with and without the elastic tensile stress (corresponding to Figure 6). Without the stress, the surface is covered by corrosion products (Figure 8B), and magnification reveals IGC (Figure 8(B1)). In contrast, the surface under stress appears smoother with fewer corrosion products (Figure 8C); however, magnification reveals a river-like crack oriented along the direction of the stress, confirming the occurrence of stress corrosion (Figure 8(C1)).

A comparison of Figure 7 and Figure 8 reveals distinct corrosion behaviors. Under no-stress conditions, IGC occurs at both low and high S_2_O_3_^2−^ concentrations, but is more pronounced in the higher-concentration solution. Conversely, when stress is applied, stress corrosion cracks are consistently observed regardless of the S_2_O_3_^2−^ concentration, indicating that stress is the dominant factor for cracking.

#### 2.4.2. Composition

**Figure 9:**
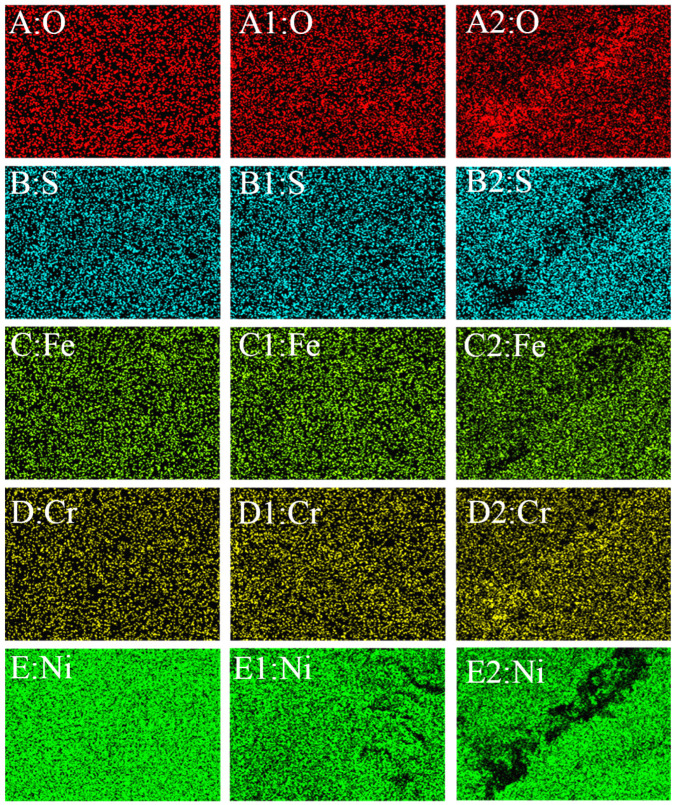
EDS results of the sample corresponding to Figure 7(A1–C1). (**A**–**E**): Blank; (**A1**–**E1**): After anodic dissolution without elastic tensile stress; (**A2**–**E2**): After anodic dissolution with elastic tensile stress. Different colors indicating varying concentrations of elements.

Figure 9 shows the EDS surface-scanning maps for the samples shown in Figure 7(A1–C1). Figure 9(A–E,A1–E1,A2–E2) represent the contents of O, S, Fe, Cr, and Ni on the surface before and after anodic dissolution, without and with elastic tensile stress, respectively. Compared with the initial blank surface, anodic dissolution without stress led to an enrichment of O, S, and Cr, and a depletion of Ni and Fe. Under stress, the crack-tip chemistry was distinct, showing higher levels of O and Cr but significantly lower levels of S, Fe, and Ni than in the no-stress condition at the crack.

**Figure 10:**
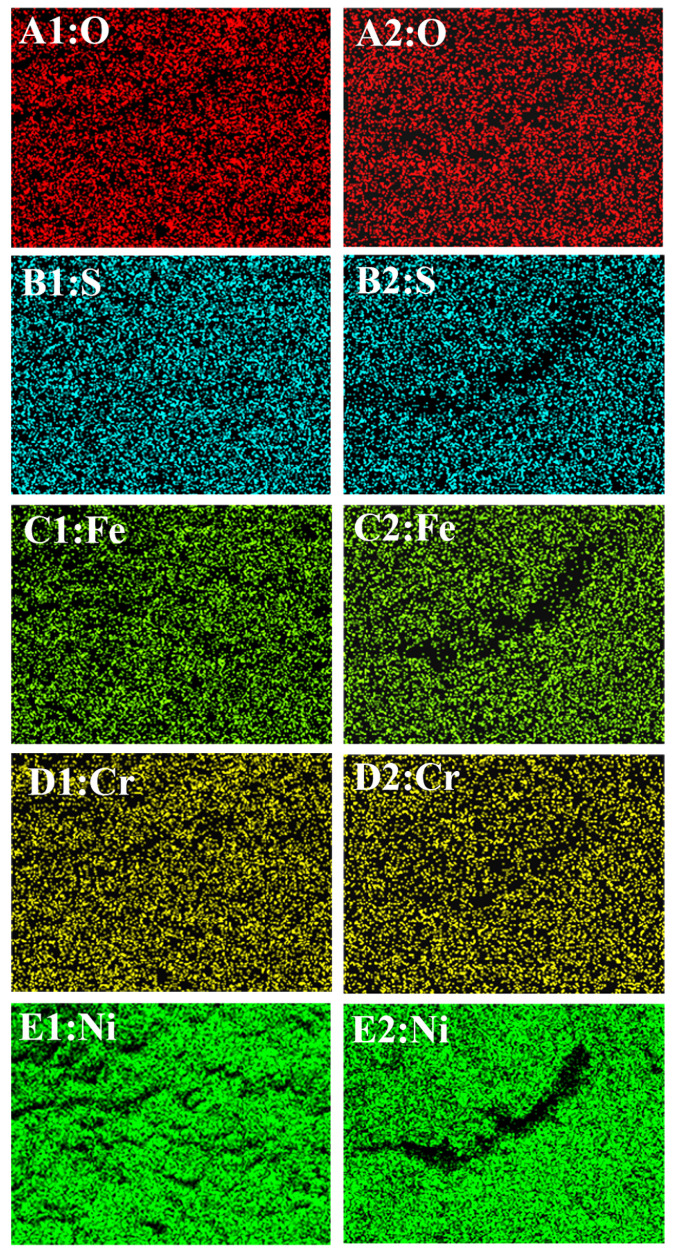
EDS results of the sample corresponding to Figure 8(B1,C1). (**A1**–**E1**): After anodic dissolution without elastic tensile stress; (**A2**–**E2**): After anodic dissolution with elastic tensile stress. Different colors indicating varying concentrations of elements.

Figure 10 shows the EDS results for the samples shown in Figure 8(B1,C1). Figure 10(A1–E1,A2–E2) represents the contents of O, S, Fe, Cr, and Ni on the surface after anodic dissolution without and with elastic tensile stress, respectively. Under stress, the crack tip showed a slightly higher O content. However, it exhibited a significant depletion of S, Cr, Fe, and Ni at the crack compared to the no-stress condition, with the loss of Ni being particularly severe.

Table 2 shows the elemental composition corresponding to the EDS results shown in Figure 9 and Figure 10. As shown in Table 2, compared with the results under stress-free conditions, the application of elastic stress leads to a decrease in Fe content, an increase in Cr content, relatively minor changes in O content, and virtually no alteration in S content. Compared to the lower-concentration solution, the decline in Ni content is significantly more pronounced in the higher-concentration solution, regardless of whether external stress is applied. This indicates that anodic dissolution of the electrode is faster in higher-concentration solutions.

## 3. Discussion

The primary objective of this discussion is to elucidate the mechanism by which low elastic tensile stress (<50% of yield strength) influences the anodic dissolution of Alloy 625 in Cl^−^ + S_2_O_3_^2−^ solutions. Given that the applied stress contributes minimal mechanical energy, its effect must be mediated through its interaction with the alloy’s surface film.

### 3.1. Stress-Induced Defects in the Oxide Film

**Figure 11:**
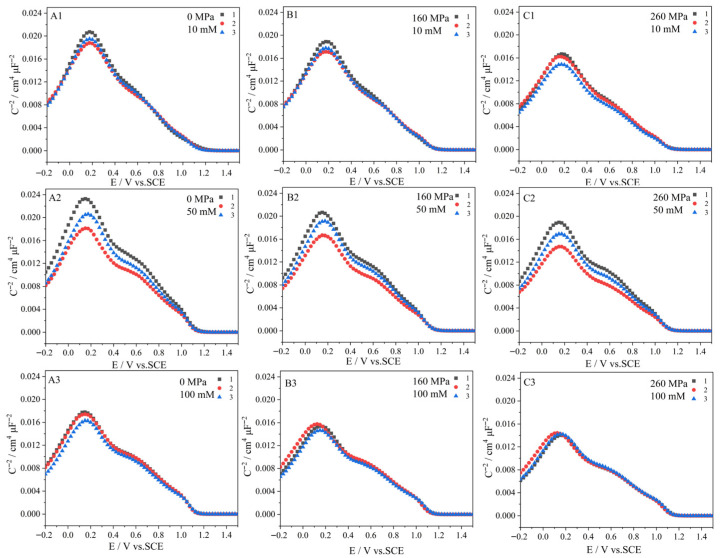
Mott–Schottky plots of passive films formed on Alloy 625 in the open air measured in 0.5 M NaCl + 10 mM Na_2_S_2_O_3_ (**A1**–**C1**), 0.5 M NaCl + 50 mM Na_2_S_2_O_3_ (**A2**–**C2**), and 0.5 M NaCl + 100 mM Na_2_S_2_O_3_ (**A3**–**C3**) solutions, with and without the elastic tensile stress, respectively, the applied frequency: 1000 Hz. Numbers 1–3 represent the number of repetitions.

We propose that the principal role of the elastic stress is to increase the defect density within the oxide film. To test this hypothesis, Mott–Schottky analysis was conducted. Figure 11 shows the Mott–Schottky plots in 0.5 M NaCl + 10 mM Na_2_S_2_O_3_ (A1–C1), 0.5 M NaCl + 50 mM Na_2_S_2_O_3_ (A2–C2), and 0.5 M NaCl + 100 mM Na_2_S_2_O_3_ (A3–C3) solutions, with and without stress, respectively. Each experiment is repeated three times. The slope donor density N_A_ can be calculated as described in the reference [23]. The calculated acceptor density (N_A_) for 0.1 V < E < 1.2 V, corresponding to the concentration of cation vacancies in the p-type semiconductor film, is summarized in Table 3. The results clearly show that the applied stress increases N_A_ in each solution. The higher the stress, the higher the acceptor density. This increase in defect density increases the corrosion current and the anodic current, as consistently observed in the Tafel curves (Figure 1 and Figure 2), Table 1, j-E (Figure 3 and Figure 4), and j-t curves (Figure 5 and Figure 6).

### 3.2. The Role of S_2_O_3_^2−^

Without the stress, the anodic current increases in the high-concentration solution (Figure 3, Figure 4, Figure 5 and Figure 6). This can be explained by two concurrent effects: (1) the adsorption of S_2_O_3_^2−^ anions themselves may increase defects in the oxide film, and (2) the high S_2_O_3_^2−^ concentration facilitates the formation of a surface salt film (Me_2_(S_2_O_3_)z) or the formation of sulfide (Me_2_S_n_) by Me (Me = Ni, Fe, Cr) and S_2_O_3_^2−^ according to Equation (2) [24], which inhibits the formation of the defective oxide film, as reflected by the higher N_A_ values (Table 3). The influence of S_2_O_3_^2−^ concentration is also the same under stress. As shown in Table 2, the Ni content decreases much more, indicating that the anodic dissolution is faster in the higher-concentration solution than in the lower-concentration solution, which is consistent with the results in Table 3.

8 Me + 6n H^+^ + n S_2_O_3_^2−^ → 2 Me_2_S_n_+ 4 Me^z+^ + 3n H_2_O (2)

The phase maps (II) in Figure 3, Figure 4, Figure 5 and Figure 6 reveal insights into the anodic dissolution processes. During these processes, soluble materials such as Me^z+^ (Me = Ni, Fe, Cr) ions are generated, increasing the concentration of Me^z+^ at the interface (inner layer). These Me^z+^ ions undergo hydrolysis and/or form a salt film with S_2_O_3_^2−^, thereby decreasing their concentration in the outer layer. In low concentrations of S_2_O_3_^2−^ solution (Figure 5), the formation of the salt film is less pronounced, resulting in a slight decrease in concentration at the interface. Conversely, at high concentrations of S_2_O_3_^2−^ solution (Figure 6), the salt film formation is more prevalent, leading to a more pronounced decrease in concentration at the interface. As shown in Figure 3, Figure 4, Figure 5 and Figure 6, the elastic tensile stress promotes the alloy’s anodic dissolution, leading to an earlier onset of localized corrosion.

### 3.3. Competing Mechanisms: IGC vs. SCC

The analysis reveals a critical shift in the dominant corrosion mechanism, depending on the stress and the thiosulfate concentration. Without applied stress, the alloy is susceptible to IGC. According to the Cr-depletion model [25,26], grain boundaries exhibit lower Cr and higher Fe/Ni concentrations than the grain matrix, making them more electrochemically active [27]. This leads to the preferential adsorption of sulfur species—generated from S_2_O_3_^2−^ decomposition—at these boundaries, inducing IGC (Figure 7(B1) and Figure 8(B1)). As the S_2_O_3_^2−^ concentration increases, IGC is significantly enhanced due to the greater availability of sulfur for adsorption.

The application of stress fundamentally alters the corrosion behavior mentioned above. The phase maps (Figure 3, Figure 4, Figure 5 and Figure 6) show that localized corrosion initiates under both conditions, suggesting that the initial stage of anodic dissolution is similar under both stressed and stress-free conditions. In the absence of stress, the process proceeds to intergranular corrosion (IGC) (Figure 7(B1) and Figure 8(B1)), whereas cracks form only in stressed samples after 150 s (Figure 7(C1) and Figure 8(C1)). It can thus be reasonably inferred that stress corrosion initiates via localized corrosion/IGC. This indicates that the stress promotes a transition from localized IGC to propagating the stress corrosion. The mechanism for this transition is twofold. First, as established, the stress creates a high density of defects in the oxide film. Second, these stress-induced defects appear to dominate the corrosion process, effectively weakening the specific role of grain-boundary chemistry.

With the applied stress, the adsorption of sulfur at grain boundaries is diminished, leading to the observed inhibition of IGC. Instead, corrosion is concentrated at the mechanically generated defects, particularly at the tip of a forming crack, where anodic dissolution of Fe and Ni is significantly enhanced. This is supported by the EDS results, which show a marked depletion of Fe and Ni (Table 2) within the cracks under stress (Figure 9 and Figure 10). The EDS data (Figure 9 and Figure 10) further support this: in the low-concentration solution, the crack tip shows enrichment of Cr and O, indicating an oxide-dominated film. In contrast, in the high-concentration solution, Cr and O are not enriched, suggesting that the rapid formation of a salt/sulfide film suppresses oxide formation and alters the local chemistry, promoting crack propagation.

## 4. Materials and Methods

Alloy 625 electrodes (C-0.04%, Si-0.27%, Cr-22.1%, Fe-4.1%, Mo-8.2%, Mn-0.36%, Ti-0.23%, Nb-3.34%, Ni-balance) were sequentially ground with #600–#2000 grit metallographic sandpaper. Subsequently, the sample was polished using a metallographic grinding and polishing machine (YMPZ-1, Shanghai metallographic machinery equipment Co., Ltd., Shanghai, China) with a 2.5 μm alumina (Al_2_O_3_) suspension, and then ultrasonically cleaned in ethanol and distilled water. A platinum sheet (10 mm × 10 mm) served as the counter electrode, and a saturated calomel electrode (SCE) as the reference electrode. A Luggin capillary minimized IR drop. All potentials are reported vs. SCE.

Figure S1 (in Supplementary Materials) shows a sketch of the tensile specimen, with a 130 mm gauge length and a 2 mm thickness. For each test, the electrode area exposed in the solution was ~ 0.3 cm^2^. The tensile stress (260 MPa, ~46% yield strength) was applied via a C-ring [23]. Electrochemical measurements (Tafel, polarization, Mott–Schottky) were performed using a CHI 660E analyzer (Chen-Hua Instrument, Shanghai, China). Tafel/polarization curves were scanned at 1 mV/s and 10 mV/s, respectively.

Mott–Schottky tests were performed in each solution with the following parameters: a frequency of 1000 Hz, a signal amplitude of 10 mV, and a potential scan from 1.2 V to −0.2 V in 25 mV steps. The results are presented as the inverse square of capacitance (1/C2) as a function of potential (E), in accordance with the Mott–Schottky relation expressed in Equation (3) [28]:

(3) $$\frac{1}{C^{2}}=-\frac{2}{\varepsilon \varepsilon_{0}{eN}_{A}}\left(E-E_{fb}-\frac{kT}{e}\right)$$

where

*N_A_*—acceptor density for a p-type semiconductor;
*ε*_0_—vacuum permittivity (8.85 × 10^−12^ F m^−1^);
*ε*—dielectric constant of the oxide film (taken as 15.6 for passive films formed on Alloy 625 [23]);
*e*—elementary charge (1.6 × 10^−19^ C);
*k*—Boltzmann constant (1.38 × 10^−23^ J K^−1^);
*T*—absolute temperature in Kelvin;
*E*—applied potential in V_SCE_;
*E_fb_*—flat-band potential.

Surface morphology/composition was analyzed using JSM-6510 SEM/EDS. Accelerating voltage, probe current, and dwell time of EDS acquisition conditions were 15 kV, 10 μA, and 200 μs, respectively. Solutions were prepared with analytical-grade reagents and distilled water. Tests were conducted at room temperature (25 ± 2 °C).

The holographic setup followed Yuan et al. [29], as shown in Figure S2 (in Supplementary Materials). The relationship between refractive index change (Δn), phase shift (ΔΦ), and concentration variation (Δc) at the interface is:

Δc = kΔn = kλ_0_ΔΦ/(2πd) (4)

where k is the concentration-refractive-index coefficient, λ_0_ is the wavelength of light, and d is the geometric path length.

A computer was employed to record in situ (as an AVI file) the dynamic changes at the electrode|electrolyte interface during an electrochemical reaction. The holograms shown here were extracted from the AVI file and converted to a color-scale representation.

## 5. Conclusions

This work combines electrochemical methods and digital holography to study the stress corrosion process of Alloy 625 in Cl^−^ + S_2_O_3_^2−^ solutions under stressed and unstressed conditions, and the following conclusions are drawn:

(1) Without the elastic tensile stress, corrosion is governed by microstructural heterogeneity, and sulfur adsorption at Cr-depleted grain boundaries drives IGC, with the severity increasing with S_2_O_3_^2−^ concentration.

(2) The applied elastic tensile stress does not simply accelerate a single corrosion process but rather increases the cation vacancy density in the oxide film, enhancing anodic dissolution and promoting stress corrosion. The high S_2_O_3_^2−^ concentration further accelerates the stress corrosion by promoting a mixed salt/sulfide surface film that facilitates crack propagation. This process simultaneously inhibits classical IGC by overriding the role of grain-boundary chemistry.

(3) Stress corrosion initiated via localized corrosion/IGC and propagated as stress-aligned cracks.

## Figures and Tables

**Table 1:** The relevant parameters of the Tafel curves shown in Figure 1 and Figure 2.

Solution	Stress/MPa	E_corr_/V	j_corr_/μA cm^−2^	−b_c_/mV
0.5 M NaCl + 10 mM Na_2_S_2_O_3_	0	−0.22	0.732	49
	260	−0.17	0.982	50
0.5 M NaCl + 100 mM Na_2_S_2_O_3_	0	−0.26	0.685	49
	260	−0.15	2.991	37

**Table 2:** Surface composition corresponding to EDS shown in Figure 9 and Figure 10.

Content (wt%)	Blank	0.5 M NaCl + 10 mM Na_2_S_2_O_3_		0.5 M NaCl + 100 mM Na_2_S_2_O_3_	
		0 MPa	260 MPa	0 MPa	260 MPa
Ni	66.91	70.57	70.74	67.89	66.41
Fe	3.84	5.12	4.71	5.03	4.47
Cr	18.24	15.97	16.62	13.33	14.3
O	0.77	1.54	1.23	4.53	3.56
S	0.21	0	0	0.18	0

**Table 3:** The acceptor densities (N_A_) with and without the stress.

c_Na2S2O3/_mM	10	50	10
N_A_ (0 MPa)/10^18^ cm^−2^	5.123 (3.41% *)	5.164 (8.82%)	6.226 (3.03%)
N_A_ (160 MPa)/10^18^ cm^−2^	5.564 (3.56%)	5.591 (7.5%)	7.073 (2.43%)
N_A_ (260 MPa)/10^18^ cm^−2^	6.297(4.66%)	6.274 (8.55%)	7.753 (0.87%)

*: The relative errors.

## Data Availability

The raw/processed data needed to reproduce these findings cannot be shared at this time, as the data also form part of an ongoing study.

## Supplementary Materials

The following supporting information can be downloaded at: https://www.mdpi.com/article/10.3390/molecules31101716/s1, Figure S1: The illustration of the test specimen. Figure S2: The experimental setup of the digital holographic interface imaging system. M: Mirror; BS: Beam Splitter; LTM: Light Transmitting Mirror; EC: Electrolytic cell; SF: Spatial filter; L1, L2 and L3: Lens.
