# Supplementary material for: In Situ Holographic Monitoring of Stress Corrosion Dynamics of Alloy 625 in Cl− + S2O32− Solution
Source: Molecules. 2026 May 18;31(10):1716. doi: 10.3390/molecules31101716 (PMC13209969; doi:10.3390/molecules31101716)
Supplement: Supplementary file 1 [file molecules-31-01716-s001.zip › molecules-4236071-supplementary.pdf]

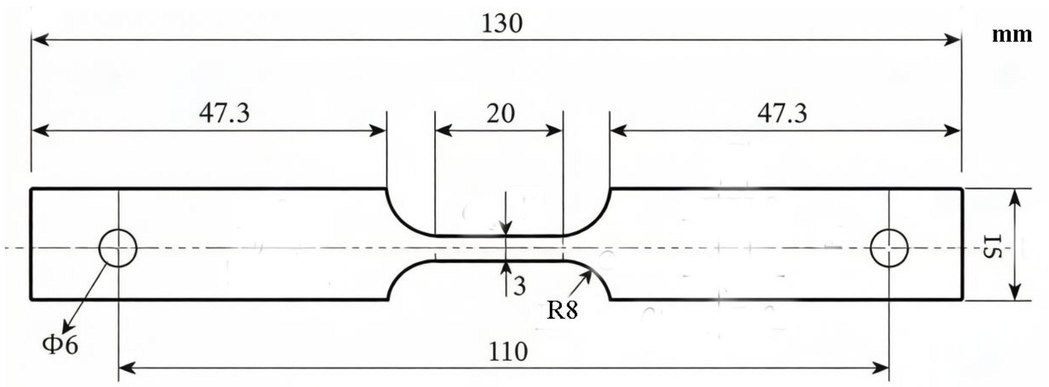

Figure S1. The illustration of the test specimen.

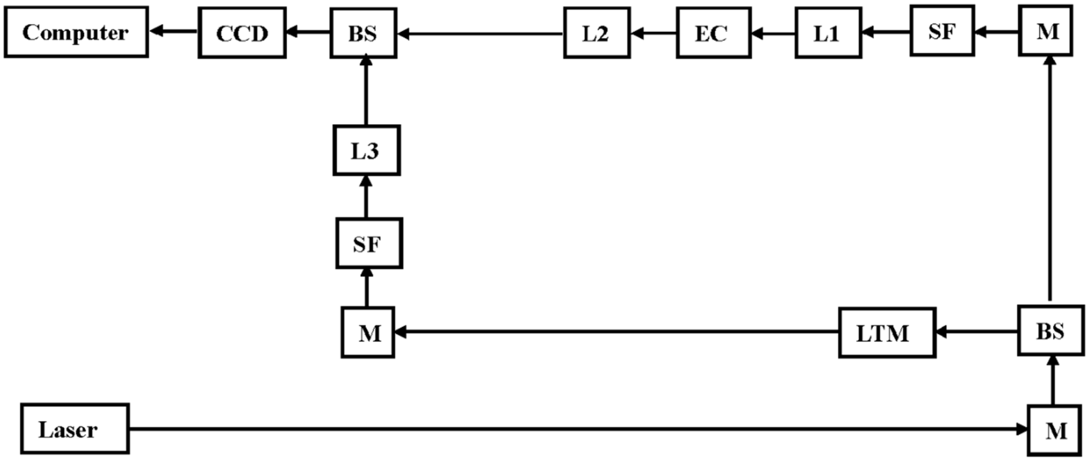

Figure S2. The experimental setup of the digital holographic interface imaging system. M: Mirror; BS: Beam Splitter; LTM: Light Transmitting Mirror; EC: Electrolytic cell Cell; SF: Spatial filter; L1, L2 and L3: Lens.
